# Radial extracorporeal shock wave therapy alters plantar pressure distribution in chronic plantar fasciitis: a biomechanical analysis

**DOI:** 10.3389/fresc.2025.1729608

**Published:** 2026-01-09

**Authors:** He Shang, Tao Ma, Yuan Wei, Tianxiang Yang, Jinpeng Liang, Xueqi Liu, Jun Li, Yi Wang, Desheng Chen

**Affiliations:** 1 The Third Clinical Medical College of Ningxia Medical University, Yinchuan, China; 2 Department of Joint Surgery, Ningxia Hui Autonomous Region People’s Hospital (Affiliated Hospital of Ningxia Medical University), Yinchuan, China; 3 Department of Pediatrics, Affiliated Hospital of Xuzhou Medical University, Xuzhou, China

**Keywords:** biomechanics, plantar fasciitis, plantar pressure, radial extracorporeal shock wave, therapeutic effects

## Abstract

**Objective:**

This study aimed to investigate the impact of radial extracorporeal shockwave therapy (rESWT) on plantar pressure distribution in patients with chronic plantar fasciitis (PF). While prior research has established rESWT's clinical efficacy in pain relief, its specific biomechanical effects on plantar loading patterns remain less quantified. This study addresses that gap by providing objective pedobarographic evidence of rESWT-induced pressure redistribution.

**Methods:**

A cohort of 42 patients with unilateral chronic PF was enrolled. Plantar pressure parameters—including total foot pressure, forefoot pressure, rearfoot pressure, peak pressure point, mean pressure, and contact area—were assessed using a plantar pressure measurement system before and after a standardized rESWT protocol. Evaluations compared the affected limb with the contralateral unaffected limb.

**Results:**

Following rESWT, significant changes were observed: on the affected side, forefoot pressure increased (*p* = 0.001) and rearfoot pressure decreased (*p* = 0.001); on the unaffected side, forefoot pressure also increased (*p* = 0.002) and rearfoot pressure decreased (*p* = 0.003). Total foot pressure decreased on the affected side (*p* = 0.032) but increased on the unaffected side (*p* = 0.032). Contact area increased significantly only on the unaffected side (*p* < 0.001). No significant alterations were found in peak pressure point location or mean pressure (*p* > 0.05). Effect size analysis (Cohen's *d* > 0.5) confirmed clinically meaningful improvements in forefoot and rearfoot pressure shifts.

**Conclusion:**

rESWT effectively ameliorates abnormal plantar pressure distribution in PF patients, promoting a forward shift in the pressure center and improving gait symmetry. These findings provide a biomechanical rationale for its clinical use. Future studies should incorporate patient-reported outcomes and longer follow-up to link these biomechanical changes to functional improvement.

## 1 Introduction

Plantar fasciitis (PF) is the most common cause of heel pain, with underlying biomechanical abnormalities contributing to microtrauma and chronic inflammation of the plantar fascia. Pathogenetically, PF develops as a consequence of repetitive trauma to the plantar fascia, leading to structural changes such as increased thickness and potential myxoid degeneration, although the exact biomechanical properties remain unclear (1, 2). The pathophysiology involves both inflammatory processes, characterized by vasodilation and immune system activation, and non-inflammatory mechanisms, including fibroblastic hypertrophy, often resulting in a chronic degenerative state rather than acute inflammation (3). This condition is frequently associated with anatomical deformities like pes planus, biomechanical factors such as excessive pronation of the subtalar joint, and chronic diseases including obesity and diabetes mellitus, with pain typically worsening after periods of rest or prolonged ambulation (4). Epidemiologically, PF affects approximately 10% of the general population and accounts for 11%–15% of foot pain visits requiring professional care, making it a leading cause of heel pain in adults, particularly among middle-aged individuals and athletic or physically active groups like conscripts (5, 6). Risk factors such as obesity, middle age, prolonged exercise, and gastrocnemius-soleus tightness significantly increase susceptibility, with higher prevalence observed in females for bilateral involvement and in cases correlated with the severity of hallux valgus deformity (7, 8).

Radial extracorporeal shock wave therapy (rESWT) has emerged as an effective treatment, alleviating pain and improving function in chronic PF through mechanisms involving tissue regeneration, anti-inflammatory effects, and biomechanical modulation. Imaging studies show reduced plantar fascia thickness post-rESWT, and biomechanical research indicates it can normalize plantar pressure distribution. Moreover, evidence from randomized controlled trials demonstrates that rESWT significantly improves pain and foot function outcomes in patients with chronic plantar fasciitis, achieving moderate to substantial relief without significant side effects (9). Comparative studies indicate that rESWT may outperform corticosteroid injections in long-term efficacy, reducing recurrence rates and enhancing functional recovery in chronic cases (10, 11). Advanced imaging techniques, such as MRI, reveal reductions in soft-tissue edema and bone marrow edema after rESWT, correlating with clinical improvements in pain and function (12). Biomechanical assessments using pedobarographic measurements confirm the normalization of plantar pressure distribution, supporting rESWT's role in addressing underlying biomechanical dysfunctions (13). Overall, rESWT is established as a safe and promising rehabilitation intervention for chronic plantar fasciitis, offering a viable alternative to conventional treatments and yielding favorable long-term results (14).

While the clinical benefits of rESWT are established, detailed quantitative analyses of its immediate impact on dynamic plantar pressure parameters remain limited. Most prior studies focus on pain scores or ultrasound findings, with fewer providing comprehensive pedobarographic data before and after intervention. This study aimed to fill this knowledge gap by systematically evaluating the effects of a standardized rESWT protocol on plantar pressure distribution in patients with unilateral chronic PF. We hypothesized that rESWT would significantly redistribute plantar pressure, characterized by a reduction in rearfoot loading and a compensatory increase in forefoot pressure, thereby improving overall gait biomechanics.

## 2 Materials and methods

### 2.1 Research subjects

A clinical evaluation was conducted on 42 patients with unilateral PF who presented at our institution between August 2024 and December 2024. Sample size was calculated based on prior similar biomechanical studies (15, 16). Using G*Power 3.1, for a paired t-test (two-tailed) with an effect size (*d*) of 0.5, alpha of 0.05, and power of 0.80, the minimum required sample size was 34. We enrolled 42 participants to account for potential dropouts. The cohort included 18 males and 24 females, with 20 left and 22 right affected limbs (Table 1). Disease duration was 5.64 ± 7.52 months, confirming chronic PF (17). All participants provided written informed consent.

**Table 1:** Demographic and clinical characteristics of participants (*n* = 42).

Characteristic	Mean ± SD/*n* (%)
Age (years)	46.98 ± 13.26
Sex (male/female)	18 (42.9%)/24 (57.1%)
Affected side (left/right)	20 (47.6%)/22 (52.4%)
Body weight (kg)	68.23 ± 11.68
Height (cm)	167.26 ± 8.94
BMI (kg/m^2^)	24.32 ± 3.22
Disease duration (months)	5.64 ± 7.52

Inclusion criteria comprised: (1) fulfillment of PF diagnostic criteria; (2) age 18–60 years; (3) BMI ≤ 30 kg/m^2^; (4) unilateral pathology; (5) symptom duration ≥3 months; (6) no prior rESWT or steroid injections within the preceding 3 months (11); (7) provision of informed consent. Exclusion criteria were: (1) foot arch abnormalities; (2) prior foot surgery; (3) contraindications to rESWT; (4) major psychiatric or cognitive disorders (10); (5) severe systemic comorbidities; (6) fractures or skin lesions in the affected foot.

### 2.2 Treatment methods

**Figure 1:**
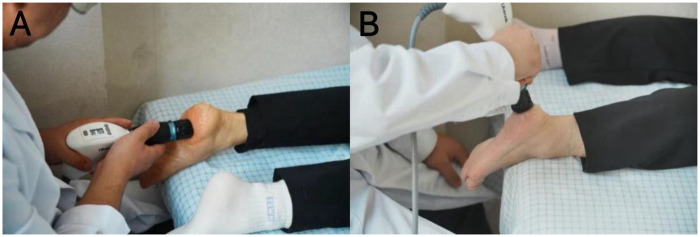
Process of rESWT treatment. **(A)** Identification of painful area; **(B)** administration of shock waves.

rESWT was administered using a Gymna ShockMaster 300 device. The painful insertion area of the plantar fascia was identified via palpation, marked, and coupled with ultrasound gel (Figure 1). Treatment parameters were standardized: frequency 8–12 Hz, intensity 2.0–3.0 Bar, starting at low energy (0.02–0.04 mJ/mm^2^) and increased to the patient's tolerable maximum, avoiding neurovascular structures (18). Each session consisted of 1,000–1,500 shocks distributed over 3–4 points within a 2 cm radius of the pain site. All participants received a fixed protocol of 4 sessions, one session per week, based on previous studies demonstrating optimal efficacy with this regimen (14). Treatments were performed by certified physiotherapists with over 5 years of musculoskeletal rehabilitation experience.

Safety Monitoring: Patients were monitored during and after each session for adverse events such as increased pain, bruising, swelling, or skin irritation. Any such events were recorded. No serious adverse events occurred; transient local discomfort was reported by some patients but resolved within 24 h (19, 20).

### 2.3 Plantar pressure detection

Dynamic plantar pressure was assessed using the Emat plantar pressure plate (3doeCom, China). All assessments were performed barefoot to avoid artifacts from footwear or socks. Data were collected pre-treatment and 1 month post-treatment completion. The testing environment was controlled (temperature 22–25 °C, humidity 40%–60%), and sessions were conducted between 9:00–11:00 AM.

Testing Procedure Explanation: Prior to testing, each participant received a standardized verbal explanation and visual demonstration of the walking protocol by a trained technician. Subjects first completed a 2-minute familiarization walk on a 10-meter walkway. For data capture, they walked at a self-selected speed (regulated to 0.8–1.0 m/s using markers) across the pressure plate. Three successful walking trials were recorded, with 1-minute rests between trials (21).

**Figure 2:**
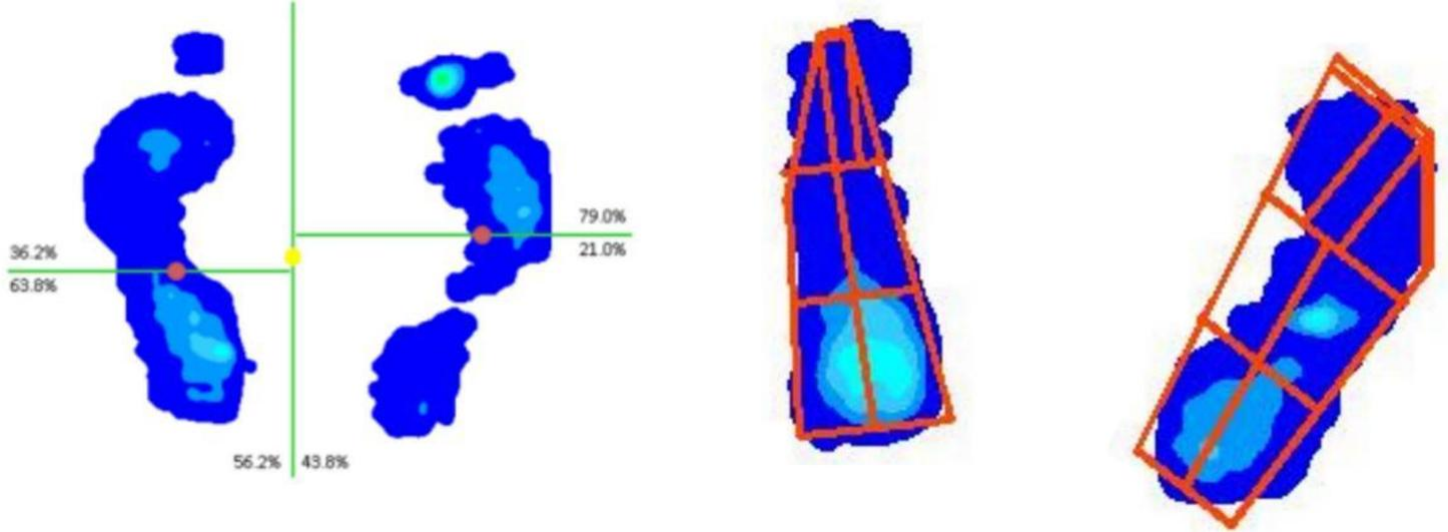
Patient foot pressure distribution map.

The pressure plate has a resolution of 4 sensors/cm^2^ and a sampling rate of 300 Hz. Data were processed using FMap software to segment the foot into forefoot, midfoot, and rearfoot regions and to extract key parameters: total foot pressure (kPa), forefoot pressure (kPa), rearfoot pressure (kPa), location of peak pressure point, mean pressure (kPa), and contact area (cm^2^) (Figure 2).

### 2.4 Statistical analysis

Data were analyzed using SPSS 26.0. Normality was tested with Shapiro–Wilk test. Paired t-tests compared pre- and post-treatment values within the same limb. Independent t-tests compared affected vs. unaffected limbs at each time point. Non-parametric alternatives (Wilcoxon, Mann–Whitney) were used if assumptions were violated. *P* < 0.05 was considered significant. Effect size was calculated using Cohen's d.

## 3 Results

Participant Flow: All 42 enrolled participants completed the 4-week rESWT protocol and the 1-month follow-up assessment. There were no dropouts or withdrawals. Therefore, all analyses were performed on the full cohort (*n* = 42) per protocol.

### 3.1 Changes in total foot, forefoot, and rearfoot pressure

Post-treatment, total foot pressure decreased significantly on the affected side and increased on the unaffected side (Table 2). Forefoot pressure increased bilaterally, while rearfoot pressure decreased bilaterally (all *p* < 0.05). Effect sizes (Cohen's *d*) for these changes exceeded 0.5, indicating moderate to large clinical effects.

**Table 2:** Changes in total foot, forefoot, and rearfoot pressure (mean ± SD, kPa).

Parameter/side	Pre-treatment	Post-treatment	*t*-value	*p*-value
Total foot pressure				
Affected	41.02 ± 11.72	35.34 ± 14.84	2.223	0.032
Unaffected	58.97 ± 11.72	64.65 ± 14.84	−2.222	0.032
Forefoot pressure				
Affected	42.42 ± 16.34	54.82 ± 18.71	−3.581	0.001
Unaffected	30.64 ± 18.06	42.91 ± 18.25	−3.222	0.002
Rearfoot pressure				
Affected	57.57 ± 16.34	45.17 ± 18.71	3.581	0.001
Unaffected	69.34 ± 18.05	57.08 ± 18.25	3.221	0.003

### 3.2 Changes in peak pressure point, mean pressure, and contact area

The location of the peak pressure point did not change significantly on either side. Peak pressure values and mean pressure showed no statistically significant changes (*p* > 0.05), though a downward trend in mean pressure was noted. Contact area increased significantly only on the unaffected side (*p* < 0.001) (Table 3).

**Table 3:** Changes in peak pressure, mean pressure, and contact area (mean ± SD).

Parameter/side	Pre-treatment	Post-treatment	*t*-value	*p*-value
Peak pressure (kPa)				
Affected	2.70 ± 1.20	2.69 ± 2.36	0.041	0.967
Unaffected	3.49 ± 1.69	3.93 ± 2.05	−1.355	0.183
Mean pressure (kPa)				
Affected	0.66 ± 0.28	0.56 ± 0.36	1.730	0.091
Unaffected	0.90 ± 0.47	0.75 ± 0.35	1.809	0.078
Contact area (cm^2^)				
Affected	49.07 ± 23.22	53.92 ± 25.20	−1.007	0.320
Unaffected	55.29 ± 24.30	70.79 ± 51.00	−3.951	<0.001

Adverse Events: As noted in Methods, no serious adverse events related to rESWT were reported. Transient local discomfort was common but self-limiting.

## 4 Discussion

rESWT has been established as an effective treatment for chronic plantar fasciitis, with numerous studies reporting significant improvements in pain relief and functional outcomes, often without adverse effects (9). In this context, our study demonstrates that rESWT significantly alters plantar pressure distribution in patients with chronic unilateral PF. The key findings are a bilateral increase in forefoot pressure, a bilateral decrease in rearfoot pressure, a reduction in total pressure on the affected side, and an increase in contact area on the unaffected side. These changes suggest a biomechanical rebalancing characterized by unloading of the painful heel and a forward shift in the pressure center during gait.

The observed reduction in rearfoot pressure on the affected side (21.5%) is clinically meaningful and aligns with the primary goal of offloading the inflamed plantar fascia insertion. The concomitant increase in forefoot pressure likely represents a compensatory mechanism to improve propulsion efficiency, a finding supported by prior gait studies (22). The fact that similar pressure shifts occurred on the unaffected side suggests a systemic biomechanical adaptation or a gait symmetry correction, where the unaffected limb adjusts its loading pattern in response to improved function on the treated side. The lack of significant change in the location of the peak pressure point indicates that rESWT does not alter the fundamental anatomical load-bearing regions but rather modulates the magnitude of load within the existing foot structure. The significant increase in contact area solely on the unaffected side may reflect a strategy to enhance stability and weight-bearing capacity during compensatory loading. These biomechanical shifts are consistent with broader evidence that ESWT enhances gait patterns and functional recovery in plantar fasciitis patients (23), and the unloading effect observed correlates with reductions in plantar fascia thickness and inflammation, as documented through objective measures like ultrasonography and MRI in other investigations (13). Furthermore, such adaptations may underlie the efficacy of rESWT in chronic cases, where it is often preferred for its role in promoting long-term symptomatic relief and structural improvements (24).

Rationale for Pressure Changes: The biomechanical effects of rESWT may be mediated through several mechanisms. By reducing pain and inflammation at the heel, it likely facilitates a more normal heel strike and weight acceptance. Furthermore, rESWT may improve the viscoelastic properties of the plantar fascia and Achilles tendon complex, allowing for better windlass mechanism function and more efficient force transfer to the forefoot during toe-off. This would explain the observed forefoot pressure increase. Our findings extend prior research by providing detailed quantitative pedobarographic evidence of these shifts, which had been suggested but less comprehensively documented. Specifically, the alleviation of pain and inflammation via rESWT, as demonstrated in controlled trials, correlates with enhanced functional outcomes and mobility, supporting its role in normalizing gait mechanics (9). Additionally, studies indicate that rESWT modifies the stiffness and elasticity of the plantar fascia, as evidenced by ultrasonographic measurements showing reduced thickness and improved tissue properties post-treatment, which contribute to optimized force distribution during activities like toe-off (25). This corroborates the biomechanical shifts toward increased forefoot pressure, with pedobarographic assessments quantitatively confirming these changes in plantar loading patterns (12).

Study Limitations and Clinical Implications: This study has limitations. First, the focus was on biomechanical parameters; we did not collect concurrent patient-reported pain or functional outcome scores (e.g., VAS, FFI). While the pressure changes provide a mechanistic rationale for clinical improvement, future studies should integrate both biomechanical and patient-centered outcomes. Second, the single-center design and modest sample size may affect generalizability (26, 27). Third, the absence of a control group (e.g., sham rESWT) means placebo effects cannot be ruled out (28). Fourth, the 1-month follow-up is short-term; longer-term durability of these pressure changes needs investigation.

Despite these limitations, our findings underscore that rESWT's therapeutic effect in PF includes a measurable normalization of foot biomechanics. For clinical practice, this supports the use of rESWT not only for pain relief but also for correcting dysfunctional gait patterns. Combining rESWT with interventions like foot orthoses or exercise therapy targeting arch strength and gait retraining may further optimize outcomes (29).

## 5 Conclusion

rESWT effectively improves plantar pressure distribution in patients with chronic plantar fasciitis, promoting a biomechanical shift from the rearfoot to the forefoot and enhancing inter-limb symmetry. These objective pedobarographic findings provide a solid biomechanical foundation for its clinical efficacy. Future research should employ controlled designs with larger samples, incorporate patient-reported outcomes, and include long-term follow-up to establish the sustainability of these biomechanical benefits and their direct correlation with functional recovery.

## Data Availability

The original contributions presented in the study are included in the article/Supplementary Material, further inquiries can be directed to the corresponding author.
